# Communication with Mechanically Ventilated Patients: Nurses’ Perspectives and Practice

**DOI:** 10.3390/nursrep15110404

**Published:** 2025-11-17

**Authors:** Ruba F. Zeadnih, Ayman M. Al-Qaaneh, Heba N. Hudhud, Islam Bani Mohammad

**Affiliations:** 1 Department of Nursing, Faculty of Nursing, Al-Balqa Applied University (BAU), Al-Salt 19117, Jordan; 2 Faculty of Allied Medical Sciences, Al-Balqa Applied University (BAU), Al-Salt 19117, Jordan

**Keywords:** mechanical ventilation, communication, communication aids for disabled, intensive care units, nursing, nonverbal communication

## Abstract

**Background**: Effective communication with mechanically ventilated patients is essential to delivering patient-centered care and ensuring psychological well-being. However, barriers such as limited verbal ability and lack of communication tools often challenge ICU nurses’ ability to interact with these patients. This study aimed to assess the communication strategies used by ICU nurses in Jordan, with a focus on their knowledge, experience, and practice regarding augmentative and alternative communication (AAC) methods. **Methods**: A cross-sectional descriptive survey was conducted among ICU nurses from 11 hospitals in Jordan (governmental, private, and military). A validated 21-item questionnaire assessed communication strategies across four domains: traditional (unaided), aided (AAC), orientation, and assessment/planning. Responses were analyzed using descriptive statistics, Friedman and Kruskal–Wallis tests. **Results**: Out of 240 invited nurses, 237 participated (98.75% response rate). Traditional unaided strategies (e.g., gestures, yes/no questions, slow speech) and assessment/planning techniques were most frequently used (median = 3.83 and 4.00, respectively), while aided AAC strategies (e.g., boards, electronic tools) were least utilized (median = 3.25). Within-group comparisons showed significant differences in communication practices across subgroups, particularly in relation to years of experience and education level (*p* < 0.05). Despite having time to listen to patients, many nurses reported lacking reliable communication methods. **Conclusions**: Jordanian ICU nurses predominantly rely on traditional communication methods when interacting with conscious mechanically ventilated patients, with limited adoption of aided AAC strategies. These findings highlight a pressing need for targeted nurse training, policy support, and improved access to AAC tools to enhance communication and patient outcomes in critical care settings.

## 1. Introduction

Mechanical ventilation (MV) is widely utilized to support critically ill patients in intensive care units (ICUs), with evidence suggesting that over 90% of ICU admissions require such support at some stage during their stay [1,2,3]. While MV is often lifesaving, it is also associated with a range of psychological and physiological consequences [4]. One of the most pervasive challenges experienced by ventilated patients is the inability to communicate, a limitation that affects more than 80% of ICU patients, often resulting in profound distress, frustration, anxiety, and a sense of powerlessness [5,6,7].

Effective communication is a fundamental human need and a cornerstone of patient-centered care. In the ICU context, nurses play a critical role in facilitating communication with mechanically ventilated patients, often needing to initiate interaction rather than wait for patient cues [8,9]. However, fulfilling this role is particularly complex, as communication barriers are common and persistent for both patients and care providers [10]. A study by Thapa et al. highlighted that 98% of mechanically ventilated patients reported communication difficulties, with many relying on hand gestures or shaking to express basic needs, while fewer than 20% used writing as a communication strategy [11].

Parallel research has explored communication with mechanically ventilated patients, either from the perspective of nurses [12] or from the perspective of patients [13,14]. These studies collectively highlight gaps in awareness and preparedness to use augmentative and alternative communication (AAC) methods. In one study involving 316 critical care nurses, approximately 30% demonstrated low awareness of AAC tools and reported an educational need for AAC-related strategies and devices [15]. The same study noted that low-tech written methods, such as paper or whiteboards, were most commonly used, despite being time-consuming. Similarly, an observational study found that nurses often deprioritized psychosocial aspects, such as communication, in favor of physiological care, potentially contributing to suboptimal treatment quality [16].

Accurate and responsive communication is not only essential for expressing patient needs and emotions but also forms the basis for evidence-based and individualized nursing care. AAC encompasses a spectrum of tools and strategies that support individuals with impaired speech or language skills [17,18,19,20,21]. These tools are categorized into low-tech (e.g., communication boards, cards), high-tech (e.g., speech-generating devices, eye-tracking systems), and no-tech (e.g., gestures, lip-reading, head nods) methods [1,18,22,23]. Studies have shown that the use of AAC can significantly reduce physiological stress markers such as blood pressure and heart rate in ventilated patients, underscoring the clinical benefit of effective communication interventions [23].

Despite the availability and proven benefits of AAC strategies, their implementation remains limited. Little is known about nurses’ knowledge, attitudes, and use of such tools in everyday practice, particularly in the context of ICU settings. Furthermore, institutional and individual barriers often hinder the adoption of these strategies. Assessing nurses’ familiarity and competency with communication techniques is therefore crucial not only to improve quality of care but also to identify systemic gaps and opportunities for targeted training and policy development.

To date, no study in Jordan has investigated the communication strategies employed by ICU nurses when caring for mechanically ventilated patients. This study aims to fill that gap by exploring Jordanian nurses’ knowledge, experience, and practices related to communication with conscious mechanically ventilated patients across various ICUs.

## 2. Materials and Methods

### 2.1. Study Design and Setting

A cross-sectional descriptive survey design was employed to evaluate the communication strategies used by nurses in intensive care units (ICUs) when interacting with conscious mechanically ventilated patients. The study was conducted across multiple ICUs in Jordan, encompassing five governmental hospitals, four private hospitals, and two military hospitals.

### 2.2. Sample Size and Sampling Technique

A purposive sampling strategy was used to recruit participants. To enhance the transferability of findings across diverse ICU settings, maximum variation sampling was applied to include nurses with differing demographic and professional characteristics. The sample size was calculated using Slovin’s formula with a margin of error set at 0.05, resulting in a minimum required sample of 225 nurses.

Inclusion criteria were: (1) nurses currently working in an ICU at one of the selected hospitals, (2) nurses with at least one year of experience caring for mechanically ventilated patients, and (3) those who provided informed consent. Exclusion criteria included: nurses in administrative roles, student or intern nurses, and individuals who declined to sign the consent form.

### 2.3. Study Instrument

Data were collected using a structured, self-administered questionnaire consisting of two sections. The first section captured participants’ demographic information. The second section assessed nurses’ communication practices through a 21-item scale covering strategies used with conscious mechanically ventilated patients. Each item was rated on a five-point Likert scale ranging from 1 (Strongly Disagree) to 5 (Strongly Agree), with higher scores indicating more frequent or favorable use of the corresponding communication strategy [23].

The instrument was originally developed by Al-Yahyai et al. to assess communication methods used by critical care nurses when interacting with severely ill or mechanically ventilated patients at Sultan Qaboos University Hospital in Oman [24]. The tool demonstrated acceptable internal consistency (Cronbach’s α = 0.78) and underwent expert validation by three specialists in critical care nursing and communication.

For the current study, the same instrument was reviewed for contextual relevance by three nursing academics in Jordan. A pilot test involving ten ICU nurses was conducted to assess feasibility and clarity; no translation was required, and only the final item was omitted based on pilot feedback due to cultural limitations. The pilot data were included in the main analysis, as no structural modifications were necessary.

The communication strategies were categorized into four subgroups:

(i) Group I: Traditional strategies (unaided methods); (ii) Group II: Augmentative and alternative strategies (aided methods); (iii) Group III: Orientation strategies (informing patients about their environment and condition); and (iv) Group IV: Assessment and planning strategies (evaluating communication ability and selecting appropriate methods).

### 2.4. Ethical Consideration and Data Collection Procedure

The study protocol was reviewed and approved by the Institutional Review Board (IRB) of Al-Balqa Applied University, Jordan (Approval No. 2025/2024/1/111) on 7 November 2024. All procedures were conducted in accordance with the ethical standards of the Declaration of Helsinki.

Following Institutional Review Board (IRB) approval, the research team met with ICU nursing administrators at each participating hospital to explain the study objectives and procedures. Eligible nurses were identified by head nurses based on inclusion criteria and were provided with an invitation letter and an information sheet describing the study purpose, voluntary nature of participation, and confidentiality assurances.

Nurses who agreed to participate provided electronic informed consent and were given a secure link to complete the self-administered questionnaire. Participation was entirely voluntary, and respondents were informed that they could withdraw from the study at any time without penalty.

To ensure anonymity and data reliability, no personal identifiers were collected, and all responses were stored in password-protected electronic files accessible only to the research team.

### 2.5. Data Analysis

Continuous variables were summarized as means ± standard deviations (SD) or medians (interquartile ranges, IQR), depending on the data distribution. The Kolmogorov–Smirnov test and Q–Q plots were used to assess data normality. As the data were not normally distributed, non-parametric statistical tests were applied.

The Mann–Whitney U test was used to assess differences in median adapted communication strategy scores between male and female participants. The Kruskal–Wallis H test evaluated differences in median scores across multiple independent groups, including years of experience, educational level, type of hospital, and working unit. To examine within-group differences across the four communication strategy domains (traditional strategies, augmented and alternative strategies, orientation of the patient to the environment, and assessment and planning of suitable strategies), the Friedman test was performed.

Whenever a significant difference was detected in any of the above tests, post hoc pairwise analyses were conducted using either the Wilcoxon Rank-Sum test (for between-group comparisons) or the Wilcoxon Signed-Rank test (for within-group comparisons). All post hoc *p*-values were adjusted using the Bonferroni correction to account for multiple comparisons. All tests were two-tailed, and a *p*-value of <0.05 was considered statistically significant. Data were analyzed using SPSS version 25.0 (IBM Corp., Armonk, NY, USA).

## 3. Results

Of the 240 nurses invited to participate in the study, 237 completed the questionnaire, yielding a response rate of 98.75%. The majority of participants were female (54.9%), with no statistically significant gender difference (χ^2^ (1, N = 237) = 2.232, *p* = 0.135). Approximately 37% of nurses reported having 1–5 years of experience caring for mechanically ventilated patients in ICUs, while 20% had 5–10 years of experience. Most respondents held a Bachelor’s degree in nursing (83.1%), and 13.5% held a postgraduate degree. Regarding institutional affiliation, 58.2% were employed in governmental hospitals, 22.4% in military hospitals, and 19.4% in private hospitals. Just over half of the participants (51.9%) worked in medical ICUs, and 88% reported caring predominantly for adult patients (Table 1).

### 3.1. Communication Strategies Utilized by ICU Nurses

Table 2 presents the various communication strategies employed by ICU nurses, expressed as mean (SD) and median (IQR). The results indicated that ICU nurses utilized a range of communication methods when caring for mechanically ventilated patients. Nearly all nurses assessed patients’ ability to communicate and developed a communication plan in collaboration with them (Group IV, Median = 4.00 [3.33–4.00]). Moreover, nurses frequently oriented patients regarding their condition, the ICU environment, date, and time. They also strived to provide reassurance by introducing themselves and emphasizing their role in supporting the patient’s recovery (Group III, Median = 3.80 [3.20–4.00]). Additionally, the vast majority of nurses relied on traditional (unaided) communication strategies, such as asking yes/no questions, recognizing patient gestures, reading lip movements, using signals and body language, and speaking slowly (Group I, Median = 3.83 [3.33–4.00]). In contrast, a smaller proportion of nurses used augmented and alternative (aided) communication strategies, including alphabet boards, picture boards, electronic devices, and writing or drawing as means of communication (Group II, Median = 3.25 [2.75–4.00]).

Table 2 reports mean and median scores for each communication domain and item. Traditional strategies showed the highest adoption (mean = 3.64 ± 0.68), followed by orientation/supportive communication (mean = 3.66 ± 0.69), and assessment/planning strategies (mean = 3.68 ± 0.79). Aided communication strategies had the lowest overall mean (3.21 ± 0.88).

### 3.2. Detailed Questionnaire Responses

Table 3 and Supplementary Figure S1 present individual item responses, showing strong agreement with traditional and supportive strategies (e.g., 59.1% agreed they speak slowly and wait for a patient’s response and 52.7% agreed they read mouthing words for mechanically ventilated patients). In contrast, aided strategies were less frequently endorsed; for instance, only 21.9% strongly agreed with using an alphabet board for communication and 5.9% strongly agreed they utilize electronic devices for facilitating communication process.

### 3.3. Statistical Comparisons by Participant Characteristics

Across all demographic subgroups, augmented and alternative strategies (Group II) were consistently the least utilized methods; this pattern was confirmed by within-group Friedman tests (all *p* < 0.001), with post hoc Wilcoxon comparisons indicating lower Group II scores than Groups I, III, and IV (Supplementary Table S1; Supplementary Figure S2A–E). For *years of experience*, between-group differences were not significant on the Kruskal–Wallis test (all *p* > 0.05). Nonetheless, within-group variability existed among nurses with <15 years of ICU experience on the Friedman test (*p* values ranging from <0.05 to <0.001), indicating heterogeneous use of strategy domains in earlier career stages (Supplementary Figure S2A; Table 4). By *educational level*, diploma-holding nurses showed no within-group differences across strategy domains (Friedman *p* = 0.538), while bachelor’s and postgraduate groups displayed significant within-group variability (*p* < 0.001 and *p* < 0.01, respectively), again driven by lower Group II scores (Supplementary Figure S2C; Supplementary Table S1). Across *hospital type*, Kruskal–Wallis tests were non-significant (all *p* > 0.05), but within-group Friedman tests were significant for governmental, private, and military hospitals (all *p* < 0.001), reflecting the same internal pattern of lower Group II usage (Table 4; Supplementary Table S1). For *working unit*, between-group differences in traditional strategies (Group I) were significant (*p* = 0.036). Bonferroni-adjusted post hoc test revealed that nurses in the Medical ICU differed significantly from those in the Surgical ICU (adjusted *p* = 0.035), with no differences for Medical vs. Cardiac or Cardiac vs. Surgical ICUs (Supplementary Table S2; Table 4).

## 4. Discussion

Communiction between nurses and mechanically ventilated patients is a cornerstone of quality critical care. Effective nurse–patient communication improves safety, satisfaction, and adherence, whereas impaired communication increases patient distress and nurses’ occupational strain [24,25,26,27]. This study evaluated the knowledge, experience, and use of augmentative and alternative communication methods by ICU nurses in Jordan, providing one of the first empirical assessments of communication practices in this context.

The present findings show that most nurses routinely assess patients’ ability to communicate and develop communication plans collaboratively (Group IV, Median = 4.00 [IQR 3.33–4.00]). This reflects a professional awareness of communication as an essential element of care, consistent with Yoo et al. (2020), who identified “recognizing communication experiences as essential for care” as a central theme among ICU nurses [28]. However, this awareness did not translate into broad use of aided AAC tools.

Traditional unaided strategies -yes/no questioning, gesture recognition, lip reading, and slow speech- were the most frequently used methods (Group I Median = 3.83). Their predominance echoes earlier work by Al-Yahyai et al. and Sias et al. (2022), who reported nurses’ reliance on simple, cost-effective interventions requiring no special training [24,29]. In resource-limited ICUs, such approaches are practical but insufficient for meeting patients’ psychosocial needs.

Aided strategies such as alphabet or picture boards and electronic devices were used least often (Group II Median = 3.25). This finding aligns with global evidence that AAC underuse stems from several inter-related causes. Handberg and Voss (2018) described how entrenched biomedical priorities and avoidance behaviors lead nurses to undervalue communication activities [16]. Additionally, low availability and poor suitability of AAC devices discourage their use (e.g., patients sedated, delirious, or visually impaired cannot easily operate boards or eye-tracking tools, reducing their perceived utility). In Jordanian ICUs, the lack of standardized aids or formal training programs, as reflected by the high agreement with the statement “no specific defined method to communicate with non-speaking patients” (Median = 4.00), reinforces this institutional barrier.

Interestingly, surgical ICU nurses used traditional methods significantly more than medical ICU nurses (adjusted *p* = 0.035). This likely reflects differences in patient profiles: surgical patients are often cognitively stable post-operation and able to respond with simple gestures, whereas medical ICU patients experience higher rates of delirium and multi-organ dysfunction [30,31]. Tailored communication training based on unit context and patient acuity could therefore enhance effectiveness.

A notable result was that two-thirds of nurses reported having time to listen patiently to patients (67.6%), contradicting the commonly cited barrier of limited time [32,33]. This suggests that the core obstacle is not lack of time but lack of structured systems -standardized AAC resources, policies, and practical education- that would allow nurses to use their existing time more effectively. Interventions should thus prioritize resource provision and competency-based training rather than workload reduction.

Collectively, these findings advance understanding of ICU communication by situating the Jordanian experience within broader international evidence. They highlight that improving nurse–patient communication requires not only equipment but also cultural and educational transformation. Embedding AAC principles into nursing curricula and hospital policy would help bridge the gap between awareness and practice.

### Study Limitations

This study has several limitations. First, its cross-sectional design captures nurses’ communication practices at a single point in time and limits causal inference. Second, data were self-reported and may be influenced by recall or social desirability bias. Third, although nurses from various hospital types were included, the findings may not be generalizable to other countries or non-ICU settings. In addition, the study did not assess attitudinal or cultural factors such as nurses’ perceptions, beliefs, or avoidance behaviors that could influence AAC use, nor did it evaluate organizational elements like tool availability, policies, or workload. The actual accessibility and suitability of AAC aids were also not documented. Future mixed-methods studies should explore how institutional resources, cultural norms, and individual attitudes interact to shape the adoption of AAC strategies in ICUs.

## 5. Conclusions

This study provides critical insights into the communication practices of ICU nurses caring for conscious mechanically ventilated patients in Jordan. While most nurses effectively utilize traditional unaided strategies and assess patients’ communication needs, the use of aided AAC methods remains limited. The findings point to a significant gap in training, resources, and institutional support that hinders the integration of AAC tools into routine practice. Addressing these barriers through structured educational interventions and policy-level reforms is essential to enhancing communication, improving patient experiences, and promoting holistic, patient-centered care in the ICU.

## Figures and Tables

**Table 1:** Demographics of the participants (n = 237).

Demographical Data (n = 237)				
Parameter		n (%)	X^2^ (*df*, N)	*p*-Value
Gender	Male (n, %)	107 (45.1%)	X^2^ (1, 237) = 2.232	0.135 ^a^
	Female (n, %)	130 (54.9%)		
Years of experience	<1	24 (10.1%)	X^2^ (5, 237) = 81.203	< 0.001 ***^a^
	1–≤5	88 (37.1%)		
	>5–≤10	46 (19.4%)		
	>10–≤15	32 (13.5%)		
	>15–≤20	22 (9.3%)		
	>20	25 (10.5%)		
Educational level	Diploma	8 (3.4%)	X^2^ (2, 237) = 268.025	<0.001 ***^a^
	Bachelor	197 (83.1%)		
	Postgraduate	32 (13.5%)		
Working hospital	Governmental	138 (58.2%)	X^2^ (2, 237) = 66.405	<0.001 ***^a^
	Private	46 (19.4%)		
	Military	53 (22.4%)		
Working unit	Medical ICU	123 (51.9%)	X^2^ (2, 237) = 36.789	<0.001 ***^a^
	Surgical ICU	56 (23.6%)		
	Cardiac ICU	58 (24.5%)		

^a^ Pearson Chi-Square test. *** *p* < 0.001 is statistically extremely significant.

**Table 2:** Questionnaire response (n = 237).

No	Question	Mean (SD)	Median (IQR)
**Group I: Traditional Strategies (Unaided Strategies)**		**3.64 (0.68)**	**3.83 (3.33–4.00)**
1	I usually use Yes/No questions in communication	3.46 (0.97)	4.00 (3.00–4.00)
2	I usually notice Patient pointing/gesturing as a method for communication	3.61 (0.93)	4.00 (3.00–4.00)
3	I usually try to read patient’ mouthing words	3.57 (0.98)	4.00 (3.00–4.00)
4	I usually use signals to communicate with non-speaking critically ill patient such as thump up for yes, shake head for No, use OK, or point to body parts.	3.73 (1.02)	4.00 (3.00–4.00)
5	I usually use body movement; fist for no, pointing, oral sounds to communicate with patients	3.56 (0.94)	4.00 (3.00–4.00)
6	I usually speak slowly and wait for patient’s response	3.90 (0.89)	4.00 (4.00–4.00)
**Group II: Augmented and alternative strategies (aided strategies)**		**3.21 (0.88)**	**3.25 (2.75–4.00)**
7	I usually use alphabet board to facilitate communication	3.26 (1.25)	3.00 (2.00–4.00)
8	I usually use picture board to facilitate communication	3.08 (1.06)	3.00 (2.00–4.00)
9	I usually Write or draw to facilitate communication	3.35 (1.01)	4.00 (3.00–4.00)
10	I usually use electronic devices to facilitate communication	3.15 (1.04)	3.00 (2.00–4.00)
**Group III: Orientation of the patient to the environment and some other information**		**3.66 (0.69)**	**3.80 (3.20–4.00)**
11	I usually tell patient about his condition and why he is unable to speak	3.75 (0.93)	4.00 (3.00–4.00)
12	I usually encourage patients by telling them that they are, e.g., doing well and/or I am helping them to get better	3.75 (0.96)	4.00 (3.00–4.00)
13	I usually introduce myself to non-speaking critically ill patients	3.70 (0.96)	4.00 (3.00–4.00)
14	I usually orient non-speaking critically ill patients to unit/environment	3.47 (0.96)	4.00 (3.00–4.00)
15	I usually orient non-speaking critically ill patients to date and time	3.62 (0.93)	4.00 (3.00–4.00)
**Group IV: assessment of communication ability and planning for suitable strategies**		**3.68 (0.79)**	**4.00 (3.33–4.00)**
16	I usually assess patients for their communication ability	3.76 (0.89)	4.00 (3.00–4.00)
17	I usually have communication plan for my patient.	3.68 (0.92)	4.00 (3.00–4.00)
18	I collaborate with non-speaking critically ill patients in choosing a communication method	3.60 (0.93)	4.00 (3.00–4.00)

19	I usually have time to listen patiently to what the patient say	3.72 (1.02)	4.00 (3.00–4.00)
20	No specific defined method to communicate with non-speaking patient in ICUs	3.57 (0.89)	4.00 (3.00–4.00)

**Table 3:** Detailed questionnaire response (n = 237).

	Questionnaire Response (n = 237)					
No.	Question	Strongly Disagree N (%)	Disagree N (%)	Neutral N (%)	Agree N (%)	Strongly Agree N (%)
1	I usually use Yes/No questions in communication	13 (5.5)	22 (9.3)	65 (27.4)	117 (49.4)	20 (8.4)
2	I usually notice Patient pointing/gesturing as a method for communication	7 (3)	20 (8.4)	64 (27)	114 (48.1)	32 (13.5)
3	I usually try to read patient’ mouthing words	9 (3.8)	29 (12.2)	46 (19.4)	125 (52.7)	28 (11.8)
4	I usually use alphabet board to facilitate communication	20 (8.4)	49 (20.7)	69 (29.1)	47 (19.8)	52 (21.9)
5	I usually use picture board to facilitate communication	18 (7.6)	54 (22.8)	71 (30)	78 (32.9)	16 (6.8)
6	I usually Write or draw to facilitate communication	11 (4.6)	38 (16)	67 (28.3)	98 (41.4)	23 (9.7)
7	I usually use electronic devices to facilitate communication	18 (7.6)	45 (19.0)	71 (30.0)	89 (37.6)	14 (5.9)
8	I usually tell patient about his condition and why he is unable to speak	7 (3)	15 (6.3)	51 (21.5)	121 (51.1)	43 (18.1)
9	I usually encourage patients by telling them that they are, e.g., doing well and/or I am helping them to get better	8 (3.4)	14 (5.9)	56 (23.6)	110 (46.4)	49 (20.7)
10	I usually introduce myself to non-speaking critically ill patients	9 (3.8)	14 (5.9)	57 (24.1)	115 (48.5)	42 (17.7)
11	I usually orient non-speaking critically ill patients to unit/environment	10 (4.2)	27 (11.4)	65 (27.4)	112 (47.3)	23 (9.7)
12	I usually orient non-speaking critically ill patients to date and time	9 (3.8)	19 (8.0)	53 (22.4)	128 (54.0)	28 (11.8)
13	I usually assess patients for their communication ability	8 (3.4)	9 (3.8)	55 (23.2)	126 (53.2)	39 (16.5)
14	I usually have communication plan for my patient.	4 (1.7)	25 (10.5)	51 (21.5)	119 (50.2)	38 (16)
15	I collaborate with non-speaking critically ill patients in choosing a communication method	11 (4.6)	14 (5.9)	59 (24.9)	127 (53.6)	26 (11.0)
16	I usually use signals to communicate with non-speaking critically ill patient such as thump up for yes, shake head for No, use OK, or point to body parts.	11 (4.6)	19 (8.0)	42 (17.7)	117 (49.4)	48 (20.3)
17	I usually use body movement; fist for no, pointing, oral sounds to communicate with patients	9 (3.8)	24 (10.1)	55 (23.2)	124 (52.3)	25 (10.5)
18	I usually speak slowly and wait for patient’s response	8 (3.4)	9 (3.8)	31 (13.1)	140 (59.1)	49 (20.7)
19	I usually have time to listen patiently to what the patient say	14 (5.9)	9 (3.8)	54 (22.8)	112 (47.3)	48 (20.3)
20	No specific defined method to communicate with non-speaking patient in ICUs	7 (3.0)	20 (8.4)	64 (27.0)	123 (51.9)	23 (9.7)

**Table 4:** Analysis based on different communication strategies (n = 237).

Analysis 237. n = 237.							
Parameter		Group I Median (IQR)	Group II Median (IQR)	Group III Median (IQR)	Group IV Median (IQR)	Within-Group Comparison	
						X^2^ (*df*, N)	*p*-Value ^c^
Gender	Male	3.83 (3.33–4.00)	3.25 (2.50–4.00)	3.80 (3.20–4.00)	3.67 (3.33–4.00)	X^2^ (3, 107) = 21.57	<0.001 ***
	Female	3.83 (3.33–4.00	3.25 (2.75–3.81)	3.80 (3.20–4.00)	4.00 (3.33–4.00)	X^2^ (3, 130) = 44.22	<0.001 ***
	**U**	6755.500	6786.000	6560.000	6505.000		
	**Z**	−0.383	−0.323	−0.758	−0.877		
	***p*-value** ^a^	0.702	0.747	0.449	0.381		
Years of experience	<1	3.75 (3.33–4.13)	3.25 (3.00–3.50)	3.80 (3.40–4.00)	4.00 (3.42–4.33)	X^2^ (3, 24) = 19.95	<0.001 ***
	1–≤5	3.83 (3.33–4.00)	3.50 (2.75–4.00)	3.80 (3.20–4.00)	4.00 (3.33–4.00)	X^2^ (3, 88) = 14.53	<0.01 **
	>5–≤10	3.83 (3.33–4.00)	3.25 (2.69–4.00)	3.80 (3.35–4.00)	4.00 (3.33–4.00)	X^2^ (3, 46) = 10.18	<0.05 *
	>10–≤15	3.83 (3.13–4.00)	3.25 (2.75–3.94)	3.90 (3.10–4.20)	3.67 (3.00–4.00)	X^2^ (3, 32) = 16.97	<0.001 ***
	>15–≤20	3.67 (3.13–3.88)	3.00 (2.19–3.50)	3.40 (3.00–4.00)	3.83 (3.00–4.00)	X^2^ (3, 22) = 6.42	0.093
	>20	3.83 (3.42–4.00)	3.50 (2.50–4.13)	3.80 (3.50–4.10)	4.00 (3.50–4.17)	X^2^ (3, 25) = 6.49	0.090
	**H**	3.184	8.248	4.319	3.693		
	**Df**	5	5	5	5		
	***p*-value** ^b^	0.672	0.143	0.504	0.594		
Educational level	Diploma	3.83 (3.00–4.00)	3.63 (2.88–4.19)	3.60 (3.40–3.80)	4.00 (3.17–4.00)	X^2^ (3, 8) = 2.17	0.538
	Bachelor	3.83 (3.33–4.00)	3.25 (2.75–3.75)	3.80 (3.20–4.00)	4.00 (3.33–4.00)	X^2^ (3, 197) = 56.01	<0.001 ***
	Postgraduate	3.83 (3.71–4.33)	3.25 (2.75–4.25)	4.00 (3.40–4.55)	4.00 (3.67–4.33)	X^2^ (3, 32) = 12.18	<0.01 **
	**H**	5.321	1.728	5.143	2.071		
	**Df**	2	2	2	2		
	***p*-value** ^b^	0.070	0.421	0.076	0.355		
Working hospital	Governmental	3.67 (3.33–4.00)	3.25 (2.50–3.81)	3.60 (3.20–4.00)	4.00 (3.00–4.00)	X^2^ (3, 138) = 31.12	<0.001 ***
	Private	3.83 (3.33–4.04)	3.38 (2.75–4.00)	3.80 (3.15–4.25)	4.00 (3.33–4.33)	X^2^ (3, 46) = 16.67	<0.001 ***
	Military	3.83 (3.50–4.00)	3.50 (2.88–4.00)	3.80 (3.40–4.10)	4.00 (3.50–4.00)	X^2^ (3, 53) = 19.81	<0.001 ***
	**H**	2.782	2.504	3.474	2.539		
	**Df**	2	2	2	2		
	***p*-value** ^b^	0.249	0.286	0.176	0.281		
Working unit	Medical ICU	3.83 (3.33–4.00)	3.25 (2.50–3.75)	3.60 (3.20–4.00)	4.00 (3.33–4.00)	X^2^ (3, 123) = 29.70	<0.001 ***
	Surgical ICU	4.00 (3.33–4.17)	3.25 (2.81–4.00)	3.80 (3.40–4.20)	4.00 (3.33–4.33)	X^2^ (3, 56) = 14.55	<0.01 **
	Cardiac ICU	3.67 (3.33–4.00)	3.50 (2.75–4.00)	3.80 (3.20–4.00)	4.00 (3.33–4.00)	X^2^ (3, 58) = 19.95	<0.001 ***
	**H**	6.650	2.335	2.886	2.438		
	**Df**	2	2	2	2		
	***p*-value** ^b^	0.036 *	0.311	0.236	0.295		

^a^ Mann–Whitney Test; ^b^ Kruskal–Wallis Test, ^c^ Friedman Test. Group I: Traditional strategies. Group II: Augmented and alternative strategies. Group III: Orientation of the patient to the environment and some other information. Group IV: assessment of communication ability and planning for suitable strategies. * *p* < 0.05 is statistically significant; ** *p* < 0.01 is statistically very significant; *** *p* < 0.001 is statistically extremely significant.

## Data Availability

The original contributions presented in this study are included in the article/Supplementary Material. Further inquiries can be directed to the corresponding author.

## Supplementary Materials

The following supporting information can be downloaded at: https://www.mdpi.com/article/10.3390/nursrep15110404/s1, Figure S1. Detailed questionnaire response. Figure S2. (A). years of experience comparison within each group. (B). gender comparison within each group. (C). educational level comparison within each group. (D). working hospital comparison within each group. (E). working unit comparison within each group. Table S1. Group comparison (post-hoc analysis) based on different communication strategies and demographics. Table S2. Pairwise Comparisons of Working unit.

## Public Involvement Statement

Nurses participated as respondents in this study; however, members of the public (patients, consumers, or careers) were not involved in the design, conduct, reporting, or dissemination of this research.

## Guidelines and Standards Statement

This manuscript was drafted according to the STROBE (Strengthening the Reporting of Observational Studies in Epidemiology) reporting guideline for cross-sectional studies.
