# Supplementary material for: Communication with Mechanically Ventilated Patients: Nurses’ Perspectives and Practice
Source: Nurs Rep. 2025 Nov 17;15(11):404. doi: 10.3390/nursrep15110404 (PMC12655519; doi:10.3390/nursrep15110404)
Supplement: Supplementary file 1 [file nursrep-15-00404-s001.zip › nursrep-3951280-supplementary.pdf]

Supplementary Figure S1

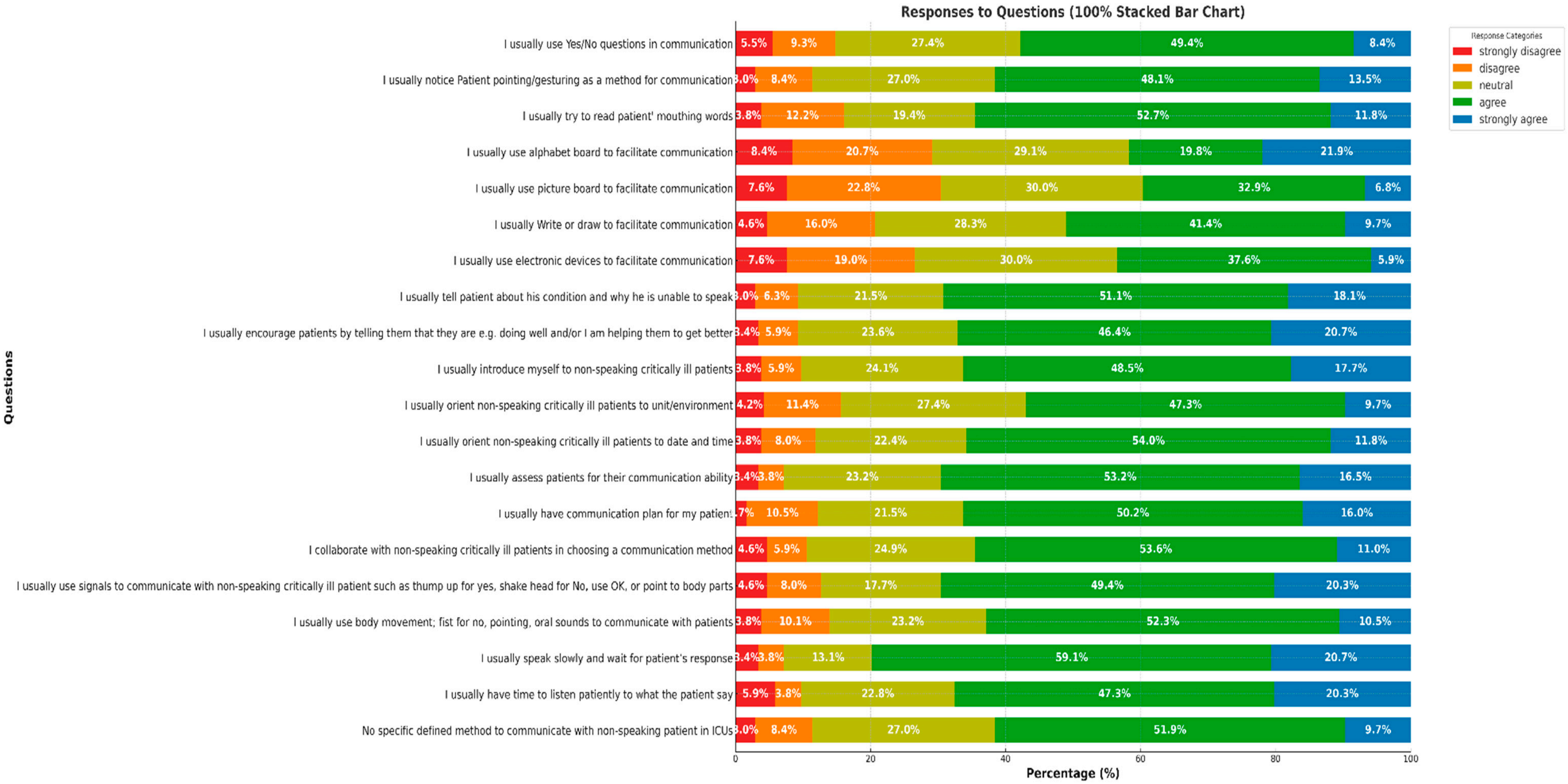

Figure S1. Detailed questionnaire response.

Supplementary Figure S2A-E

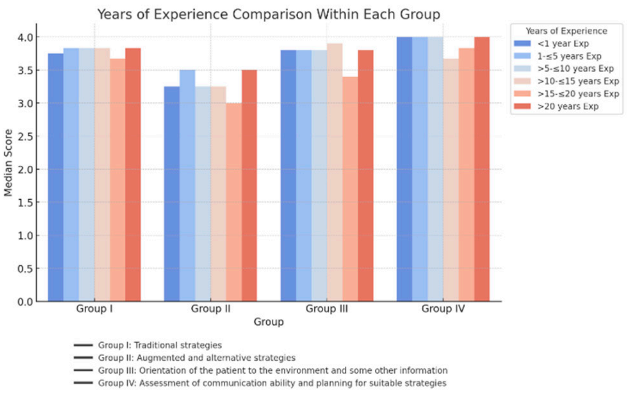

Figure S2A: years of experience comparison within each group

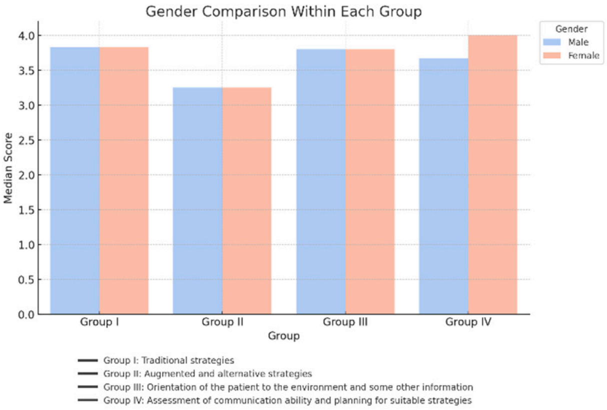

Figure S2B: gender comparison within each group

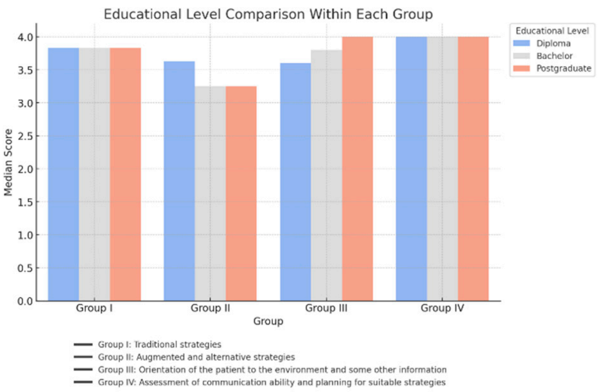

Figure S2C: educational level comparison within each group

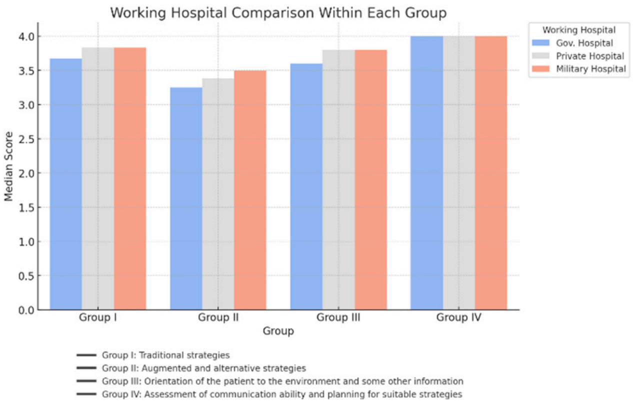

Figure S2D: working hospital comparison within each group

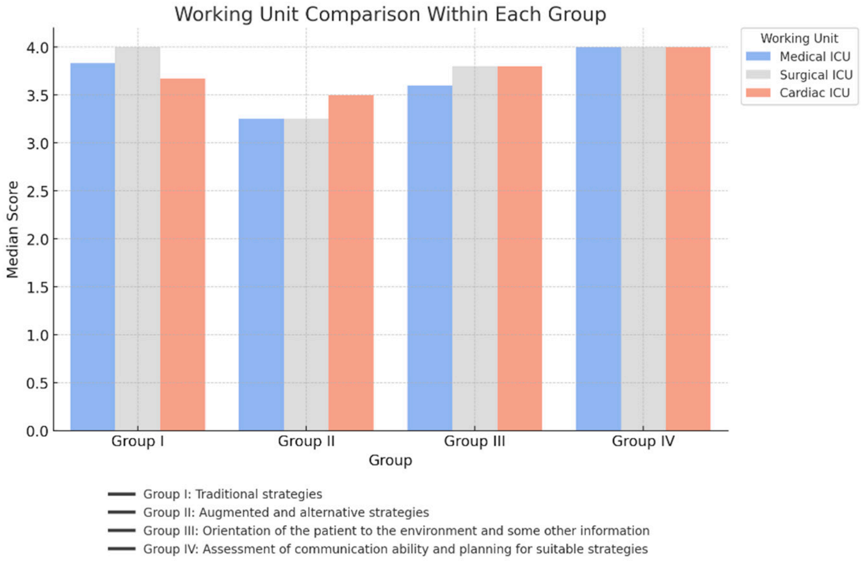

Figure S2E: working unit comparison within each group

Supplementary Table S1

Table S1: Group comparison (post-hoc analysis) based on different communication strategies and demographics[a, b] (n= 237)

Analysis based on different communication strategies (n= 237)								
**Parameter**		**Group I-Group II**	**Group I-Group III**	**Group I-Group IV**	**Group II-Group III**	**Group II-Group IV**	**Group III-Group IV**	**p-value[c]**
Gender	Male	0.000	1.000	1.000	0.000	0.000	1.000	<0.001***
	Female	0.000	1.000	0.355	0.000	0.000	1.000	<0.001***
Years of experience	<1	0.006	1.000	0.604	0.003	0.001	0.547	<0.001***
	1 - ≤5	0.005	1.000	1.000	0.005	0.000	1.000	<0.01**
	>5 - ≤10	0.001	1.000	1.000	0.001	0.001	1.000	<0.05*
	>10 - ≤15	0.069	0.251	1.000	0.002	0.026	0.257	<0.001***
	> 15 - ≤20	---	---	---	---	---	---	.093
	>20	---	---	---	---	---	---	.090
Educational level	Diploma	---	---	---	---	---	---	.538
	Bachelor	0.000	1.000	0.790	0.000	0.000	1.000	<0.001***
	Postgraduate	0.023	0.789	1.000	0.002	0.029	1.000	<0.01**
Working hospital	Governmental	0.000	1.000	1.000	0.000	0.000	1.000	<0.001***
	Private	0.001	1.000	1.000	0.014	0.001	1.000	<0.001***
	Military	0.011	1.000	1.000	0.001	0.000	1.000	<0.001***
Working unit	Medical ICU	0.000	1.000	1.000	0.000	0.000	1.000	<0.001***
	Surgical ICU	0.022	1.000	0.235	0.003	0.001	1.000	<0.01**
	Cardiac ICU	0.001	1.000	1.000	0.000	0.017	1.000	<0.001***

[a] Post-hic analysis using Wilcoxon Signed-Rank Test adjusted by the Bonferroni correction for multiple tests, [b] Probabilities with p-value more than 1 are capped to 1, [c] Friedman Test

Group I: Traditional strategies

Group II: Augmented and alternative strategies

Group III: Orientation of the patient to the environment and some other information

Group IV: assessment of communication ability and planning for suitable strategies

* p<0.05 is statistically significant; **p<0.01 is statistically very significant; ***p<0.001 is statistically extremely significant

Supplementary Table S2

Table S2: Pairwise Comparisons of Working unit

Sample 1-Sample 2	Test Statistic	Std. Error	Std. Test Statistic	Sig.	Adj. Sig.[a]
Medical ICU -Cardiac ICU	-3.353	10.841	-.309	.757	1.000
Medical ICU -Surgical ICU	-27.718	10.971	-2.526	.012	.035
Cardiac ICU-Surgical ICU	24.365	12.751	1.911	.056	.168

Each row tests the null hypothesis that the Sample 1 and Sample 2 distributions are the same.
Asymptotic significances (2-sided tests) are displayed. The significance level is .050.

a. Significance values have been adjusted by the Bonferroni correction for multiple tests.
